# Clinical Practice Guidelines for Topical NSAIDs in the Treatment of Sports Injuries

**DOI:** 10.1111/jebm.12661

**Published:** 2025-01-10

**Authors:** Yan Xiong, Yan Liu, Jingbin Zhou, Xiliang Shang, Hongchen He, Guoping Li, Shiyi Chen, Jian Li

**Affiliations:** ^1^ Department of Orthorpedics and Sports Medicine, Orthopedic Research Institute, West China Hospital Sichuan University Chengdu China; ^2^ Department of Rehabilitation Medicine, Rehabilitation Key Laboratory of Sichuan Province, West China Hospital/West China Tianfu Hospital Sichuan University Chengdu China; ^3^ Sports medicine and Rehabilitation department, Beijing Chaoyang Hospital Capital Medical University Beijing China; ^4^ Institute of Sports Medicine Huashan Hospital Affiliated with Fudan University Shanghai China; ^5^ Department of Rehabilitation Medicine, Rehabilitation Key Laboratory of Sichuan Province,West China Hospital Sichuan University Chengdu China; ^6^ Institute of Sports Medicine General Administration of Sport of China Beijing China

**Keywords:** clinical practice guidelines, sports injury, topical NSAIDs

## Abstract

Topical nonsteroidal anti‐inflammatory drugs (NSAIDs) are commonly used to treat sports injuries, but evidence‐based medical guidance for their standardized and rational use is lacking. This guideline working group identified clinically important issues, obtained the full opinions of patients and clinical staff, and discussed them with the expert group. Based on evidence from the literature, the “clinical practice guidelines for topical NSAIDs in the treatment of sports injuries” were formulated following the methods and principles of international guidelines. According to these guidelines, 7 clinical concerns were ultimately selected, and 22 recommendations were formed. These included the status, indications, contraindications, efficacy, combined application, use in special populations, adverse reactions, and countermeasures of topical NSAIDs in the treatment of sports injuries. The purpose of these guidelines is to provide evidence‐based recommendations for practitioners in the fields of orthopedics, sports medicine, rehabilitation medicine, and sports science, as well as other fields, in the treatment of sports injuries to promote more standardized and rational use of topical NSAIDs.

## 1 Background

### 1.1 Background and Purpose of the Guidelines

With the implementation of the “Healthy China” national strategy, national fitness sports are booming, and various types of sports injuries are becoming increasingly common [1]. Topical NSAIDs have been widely used to treat sports injury‐related diseases to reduce inflammation, relieve pain, and improve function [2, 3]. However, the relative proportion of clinical use of topical NSAIDs is low, treatment standardization is insufficient, and the understanding of topical NSAIDs among clinical medical staff needs to be improved, especially in terms of indications, dosage form selection, combination with other treatment schemes, and use in special populations.

In 2016, sports medicine and orthopedics experts formulated the “Chinese expert consensus on topical nonsteroidal anti‐inflammatory drugs for musculoskeletal system pain” [4], which effectively promoted the application of topical NSAIDs in the treatment of pain caused by musculoskeletal system injury. With developments in various disciplines, improvements in topical NSAID preparations, and updates to evidence‐based medical guidance, based on expert consensus work in 2016, the Chinese Society of Sports Medicine, the Chinese Orthopedic Association, the Chinese Society of Physical Medicine and Rehabilitation, the China Sports Society of Sports Medicine, the China Medicine Education Association, and the Beijing Research Association for Chronic Disease Control and Health Education jointly initiated the development of Clinical Practice Guidelines for Topical NSAIDs in the Treatment of Sports Injuries. The purpose of these guidelines is to provide evidence‐based knowledge for practitioners of orthopedics, sports medicine, rehabilitation medicine, and sports science in the treatment of sports injuries and to promote a more standardized and rational use of topical NSAIDs.

### 1.2 Definition, Diagnosis, Classification, and Epidemiology of Sports Injuries

Sports injuries involve damage to tissue structures (e.g., bone joints, muscles, tendons, ligaments, fascia, the synovium and adjacent blood vessels, nerves) due to trauma from sports or overuse due to repeated mechanical exercise, resulting in corresponding clinical symptoms and signs [5]. The diagnosis of sports injuries includes an inquiry history, physical examination, and laboratory and imaging examinations. Different diseases are classified and diagnosed by stage and, according to the injury site or tissue structure, can be classified as soft tissue injuries, including injuries to the muscle, tendon, fascia, ligament, or bursa; bone and cartilage injuries, such as osteoarthritis, stress fracture, or bone contusion; or peripheral nerve or blood vessel injuries. Sports injuries are also categorized as acute or chronic according to when the injury occurred and the duration of pain [6, 7]. Acute and chronic injuries to soft tissues, such as tendons and ligaments, are usually limited to 6 weeks, and bone and cartilage injuries can last for approximately 3 months [8, 9, 10, 11].

The reported incidence of sports injuries in China is approximately 10%–20%, which is similar to that reported in Europe and America [12]. Epidemiological studies of sports injuries have focused on specific sports, specific populations, and specific injury types [13, 14, 15]. In 2010, one survey revealed that the incidence of sports injuries in amateur sports activities of community residents in China was as high as 73.48% [16]. In 2010, a cross‐sectional study of 1239 athletes revealed an incidence of 34.9% [95% CI (29.4%, 40.4%)] for athletic injuries in men, with knee injuries occurring in 35%, shoulder injuries in 24%, and back injuries in 28%. The incidence of sports injuries in women was 45.0% [95% CI (41.5%, 48.5%)] [17]. According to WHO statistics from 2020, there are more than 300 million patients with osteoarthritis (OA) worldwide, and the overall prevalence rate of OA in people over 40 years old in China is as high as 46.3% [18, 19, 20].

### 1.3 Characteristics of Topical NSAIDs

Topical NSAIDs are topical preparations that are commonly used in clinical practice; they act directly on the skin of the injured region, penetrate through the skin to reach diseased tissues, and have anti‐inflammatory, analgesic, detumescent, and functional effects [3, 17, 21, 22]. Topical NSAIDs have the advantages of fast onset, high local concentrations, low systemic exposure, and few systemic adverse reactions. Thus, topical NSAIDs are more suitable for the treatment of sports injuries than oral NSAIDs are [23, 24, 25, 26]. Topical NSAIDs include diclofenac diethylamine, loxoprofen sodium, ibuprofen, ketoprofen, flurbiprofen, indomethacin, diclofenac sodium, etofenamate, biphenylacetic acid, and piroxicam, among other drugs, and dosage forms include patch/cream, gel, emulsion/cream, solution, and spray [27] (Table 1). Although the mechanism of action of topical NSAIDs is similar to that of oral NSAIDs, dosage forms vary, and there are also differences in clinical efficacy [28, 29, 30, 31]. An ideal topical preparation should have a low molecular weight (<500 Da), a strong effect, and both hydrophilic and hydrophobic characteristics [32]. Topical NSAID formulations usually contain three components, namely, an active ingredient, an excipient, and a transdermal absorption enhancer, which determine the clinical properties of topical NSAIDs [33]. Transdermal absorption enhancers of topical NSAIDs are added to the formulation to increase skin absorption [34]. Some excipients of topical NSAIDs, such as ethanol and glycerol, can also improve skin permeability [35]. Compared with ointments and creams, gels contain penetration aids (e.g., ethanol) that promote penetration of the skin, increasing the rate of drug delivery to the target. Gels also contain more water‐soluble ingredients, allowing more of the drug to be dissolved in the gel and facilitating drug diffusion through the skin [36]. The results of the questionnaire addressing clinical concerns in this guideline show that topical NSAIDs are favored by patients because of their convenience, comfort, safety, and effectiveness. However, these excipients can also irritate the skin, often manifesting as localized redness, rash, and itching. Topical preparations are generally prohibited due to incomplete application or affected sites; for example, the skin, eyes, and other sensitive mucous membranes can be affected by rash [37]. In addition, ethanol and propylene glycol are common allergens, and care should be taken to ask patients about their allergy history when these chemicals are used (Table 1).

**TABLE 1:** Clinical sports injury treatment summary of topical NSAIDS.

Generic name	Formulation	Indications	Contraindicated	Usage
**Diclofenac** **diethylamine**	Emulgel Gel	Muscle, tendons, and other soft tissue inflammatory pain; limbs and spinal arthritis	Nonsteroidal anti‐inflammatory drug allergies; those allergic to isopropanol or propylene glycol and other excipients; women during pregnancy; skin lesions	Apply an appropriate amount to the affected area, 3–4 times a day
**Etofenate**	Cream	Rheumatoid disease; frozen shoulder; low back pain; tenosynovitis; arthritis of extremities and spine; contusion, sprain, chronic strain of musculoskeletal, etc.	This product and other nonsteroidal anti‐inflammatory drug allergies	Apply an appropriate amount to the affected area, 1–2 g each time (5–10 cm), 3–4 times a day
**Ketone**	Liniment Gel Patch	Local pain of osteoarthritis and soft tissue injury	Aspirin asthma (non‐steroidal anti‐inflammatory drug induced asthma), this product, or other nonsteroidal anti‐inflammatory drug allergies; patients with bleeding ulcer of stomach and duodenum	1–3 mL liniment each time, 2–3 times a day; 1 g gel each time, 3–4 times a day; 2 patch a day
**Ibuprofen**	Liniment Cream Gel	Local soft tissue pain; the pain caused by different kinds of sprain and strain, the strain; osteoarthritis	Ibuprofen or supplementary material in any of the ingredients (alcohol, propylene glycol, etc.) are allergic; aspirin or another nonsteroidal anti‐inflammatory drug allergy	Apply an appropriate amount to the affected area 3–4 times a day. Pediatric patient please consult physician or pharmacist
**Piroxicam**	Liniment Gel	Rheumatoid arthritis; osteoarthritis and frozen shoulder; tenosynovitis; lumbar muscle strain; pain and swelling from strains, sprains of sports injuries	Nonsteroidal anti‐inflammatory drugs allergies; alcohol or propylene glycol allergy	Apply an appropriate amount on the affected skin or joint, 2 times a day.
**Flurbiprofen**	Gel Patch	Osteoarthritis; frozen shoulder; tenosynovitis; tenosynovitis, lateral epicondylitis (tennis elbow), muscle pain, traumatic swelling, pain, etc.	This product or other flurbiprofen preparations have allergy history; aspirin asthma (such as nonsteroidal anti‐inflammatory drug induced asthma) or allergies	Apply to the affected area twice a day
**Indomethacin**	Liniment Cream Gel Gel paste Cataplasm	Local soft tissue pain and osteoarthritis	Indomethacin history of allergies and asthma; renal insufficiency; skin wounds, mucous membrane and eyes; children under the age of 15; it is prohibited to continuous use more than 2 weeks	2–3 times liniment a day; an appropriate amount of gel 1–4 times a day;1.5–2 g cream, 2–3 times a day;1–2 times gel patch or cataplasm a day
**Diclofenac sodium**	Liniment Cream Gel	Relieve muscle, soft tissue and joint mild to moderate pain; osteoarthritis	Nonsteroidal anti‐inflammatory drug allergies; propylene glycol allergies	1–3 mL liniment, 2–4 times a day, a day not more than 15 mL; an appropriate amount of cream, 3 to 4 times a day; 2–4 g gel, 3 to 4 times a day, not more than 30 grams a day
**Diphenylacetic acid**	Gel	Osteoarthritis and soft tissue injury caused by swelling and pain	Allergy to this product and components; pregnant women; children under the age of 12; aspirin asthma history; Contraindicated for eyes and mucous membranes	1 g each time, 2 to 4 times a day, total amount not more than 25 grams a day (approximately 25)
**Loxoprofen sodium**	Patch Gel	Arthritis, muscle pain	Active peptic ulcer/bleeding; serious hematology was abnormal; severe liver and kidney damage; patients with severe cardiac insufficiency; coronary artery bypass surgery perioperative pain management; aspirin induced asthma and allergy history	Once a day, apply to the affected area; usage for women in the third trimester, refer to the drug guidance for pregnant and lactating women

*Note*: The information sheet of NSAIDs for topical use in this guideline is extracted from the instructions of chemical drugs (or corresponding NSAIDs for topical use) of the National Food and Drug Administration. It should be used after physician evaluation in clinical practice.

Topical NSAIDs have been reported to be safer than oral preparations. Compared with those of oral preparations, the systemic absorption of topical NSAIDs and the incidence of adverse events are lower [38]. The adverse events of topical NSAIDs are mainly local skin irritation, which is relatively mild and disappears rapidly after discontinuation [39, 40]. Notably, hypersensitivity‐like reactions (both allergic and nonallergic), due mainly to hypersensitivity to NSAIDs mediated by IgE or T lymphocyte cross‐intolerance caused by a deficiency in endogenous arachidonic acid metabolism combined with COX‐1 inhibition by NSAIDs, may occur with the use of topical NSAIDs [41]. Hypersensitivity‐like reactions are characterized mainly by urticaria, angioedema, dyspnea, asthma, and other serious adverse reactions [42]. To avoid serious adverse events, the use of topical NSAIDs is prohibited in patients who are allergic to NSAIDs and patients with aspirin‐induced asthma [43] (Table 1).

## 2 Method

### 2.1 Guideline Development Process and Methodology

#### 2.1.1 Guideline Origination and Overall Approach

The initiation and formulation of the guidelines were jointly initiated by the Chinese Society of Sports Medicine, the Chinese Orthopedic Association, the Chinese Society of Physical Medicine and Rehabilitation, the China Sport Science Society of Sports Medicine, the China Medicine Education Association, and the Beijing Research Association for Chronic Disease Control and Health Education. A total of 46 well‐known first‐line experts in medicine, health care, pharmacy, and evidence‐based medicine were recruited from the East, West, South, North, and Central Regions of China. The whole process of guideline formulation is directed by evidence‐based medicine, methodology, guideline compilation, and expert submissions. The guideline working group was established and started working in December 2022. The guidelines were formulated in strict accordance with the Chinese Medical Association Guidelines for the Formulation/Revision of Clinical Diagnosis and Treatment Guidelines in China (2022 Edition) [44], registered on the international guideline registration platform (registration number: PREPARE‐2023CN272) and publicly disclosed to the project plan. The WHO manual for guideline development was used during the development and reporting processes, and the guideline recommendations incorporated the evidence grading recommendation assessment, development and evaluation methods, namely, the grading of recommendations assessment, development and evaluation (GRADE) [45] (Tables 2 and 3), and reporting items for practice guidelines in healthcare (RIGHT) [46].

**TABLE 2:** Guide quality of evidence classification and explanation.

Grading of evidence quality	Definition
**High (A)**	The authors are very confident that the true effect lies close to that of the estimate of effect.
**Moderate (B)**	The authors are moderately confident in the effect estimate: the true effect is likely to be close to the estimate of the effect, but there is a possibility that it is substantially different.
**Low (C)**	The authors’ confidence in the effect estimate is limited: the true effect may be substantially different from the estimate of the effect.
**Very low (D)**	The authors have little confidence in the effect estimate: the true effect is likely to be substantially different from the estimate of the effect.

**TABLE 3:** Strength of recommendation and description of these guidelines.

Grading of recommendation strength	Description
**Strong (1)**	Benefits clearly outweigh risk and burdens, or vice versa
**Weak (2)**	Uncertainly in the estimates of benefits, risks, and burden; benefit, risk

#### 2.1.2 Guideline Working Group

The guideline working group consists of several groups: the Guideline Development Group, which comprehensively organizes and coordinates the promotion and implementation of all work in the guidelines; the Guideline steering group, which (1) determines the scope of this guideline, (2) establishes working groups for this guideline, (3) preliminarily constructs PICO questions [47], (4) guides and supervises the development process of this guideline, and (5) reviews and approves recommendations and the publication of this guideline; the Guideline experts group, which (1) identifies clinical problems related to the guidelines, (2) guides and assists the systematic evaluation team in completing the evidence retrieval and evaluation, (3) discusses and forms recommendations for the guidelines, and (4) guides and completes the full‐text drafting and finalization of the guidelines; the Guideline systematic review group, which does literature search, data extraction, evidence screening, and systematic review; the guideline external review group, which organized a review of the initial draft of the guidelines and proposed opinions and suggestions (Table 4).

**TABLE 4:** The composition and responsibilities of the guideline working groups.

Working group	Member	Work Content
Development group	Clinical pharmacy (1), orthopedic surgery (10), sports medicine (22), rehabilitation medicine (12)	Comprehensively organizes and coordinates the promotion and implementation of all work in the guideline.
Steering group	Orthopedic surgery (1), sports medicine (4), rehabilitation medicine (1)	(1) Determine the scope of this guideline; (2) set up working groups for this guideline; (3) preliminary construct PICO; (4) guide and supervise the development process of this guideline; and (5) review and approve recommendations and the publication of this guideline.
Expert group	Clinical pharmacy (1), orthopedic surgery (10), sports medicine (16), rehabilitation medicine (5)	(1) Identifying clinical problems related to the guidelines; (2) guiding and assisting the systematic evaluation team in completing the evidence retrieval and evaluation; (3) discussing and forming recommendations for the guideline; and (4) guiding and completing the full‐text drafting and finalization of the guideline.
Systematic review group	Guideline development methodology expert (4)	Literature search, data extraction; evidence screening, systematic review.
External review group	Orthopedic surgery (2), sports medicine (2)	Organized a review of the initial draft of the guidelines and proposed opinions and suggestions.

PICO (P—Patient; I—Intervention; C—Control; O—Outcome).

#### 2.1.3 Selection and Identification of Clinical Problems

The working group on the selection and determination of clinical problems summarized and resolved the clinical problems related to the treatment of sports injury diseases with topical NSAIDs (e.g., treatment location, indications, contraindications, use in special populations and combination drugs), as well as the effectiveness indicators (e.g., pain score, function score, clinical treatment success rate and recovery of motor ability) and adverse event reports through a literature search. Then, according to the literature search results, a questionnaire was designed to investigate the importance of clinical problems among medical staff in different hospitals in many provinces and cities nationwide. A total of 200 valid questionnaires were received to screen out the initial list of clinical problems and outcome indicators. The Guidance Steering Group, which is based on PICO principles, discusses 10 preliminary clinical questions. Two rounds of the Delphi questionnaire [48] were conducted in the selection of clinical problems, and two online and offline discussion meetings were held at the same time to summarize, classify, and standardize the expression of important clinical problems. Seven clinical problems were included in these guidelines.

#### 2.1.4 Clinical Problem Sorting and Evidence Retrieval

The systematic review team independently completed the systematic review of the literature evidence for all clinical issues. This guideline includes systematic evaluation, meta‐analysis, and randomized controlled trials (RCTs) on the efficacy, safety, and adverse events of topical NSAIDs in the treatment of sports injuries. When RCT evidence is insufficient, nonrandomized controlled clinical study evidence will be added; for some clinically important issues, basic study results will be carefully supplemented as indirect evidence according to the opinions of expert groups. The literature search included the MEDLINE, Embase, Web of Science, Cochrane Library, CNKI, WanFang Data, and CBM databases up to June 2023. Chinese and English search keywords included sports injuries, sports system injuries (including specific disease names, such as osteoarthritis, tenosynovitis, lateral epicondylitis, frozen shoulder, and tenosynovitis), topical NSAIDs (including specific drugs and dosage types), treatment, special populations (including elderly people, children, pregnant women, etc.) and adverse reactions, among other items, and keyword combination was performed according to the search requirements.

#### 2.1.5 Evaluation of Evidence and Recommendation Formation

The systematic evaluation team sifted through titles, abstracts, and full‐text records for potentially relevant studies and then used standardized data extraction tables to extract data from the included studies. The systematic evaluation team cross‐checked the screening results and data extraction tables and organized expert discussions to resolve differences through the Guidelines Working Group. After the evidence search was completed, the systematic review team performed quality assessment and evidence integration. For systematic review and meta‐analysis, a measurement tool to assess systematic reviews (AMSTAR) was used; for RCTs, the risk of bias (RoB) was used to assess potential bias risk. When there were disputes on the evaluation of study bias, such as selection bias, detection bias, and depletion bias, the guideline working group organized experts to discuss and reach a consensus. A total of 200 valid questionnaires were received based on patient preferences and values. The secretary group summarized and analyzed the results and fed them back to the guideline formulation group and guidance group to provide a reference for the formation of recommendations. Finally, the direction and intensity of the recommendations were established via the Delphi method, and an expert group discussion was held on November 11, 2023, to discuss and vote on the recommendations. The recommendations were finalized and voted on December 29, 2023, and 22 recommendations were ultimately developed for the guidelines.

### 2.2 Guidelines for Use and Target Populations

This guideline applies to medical and healthcare facilities at all levels. The target users included clinicians (including doctors in orthopedics, sports medicine, rehabilitation medicine, pain management, general practice, emergency departments, etc.) and licensed practitioners engaged in disease prevention and health care related to sports injuries. The target patient population was patients with acute or chronic sports injuries.

### 2.3 Update and Dissemination of Guidelines

This guideline is published preferentially in academic journals, and opinions on the interpretation and promotion of the guideline guide are written. Moreover, the guideline working group plans to update the guidelines every three to five years. Once published, the guidelines are disseminated, for example, through academic conferences or training courses. The specific modes of dissemination include the following: ① dissemination at multidisciplinary academic conferences such as sports medicine, orthopedics, rehabilitation medicine, and sports science; ② publication and dissemination of the guideline text in the form of academic journals, monographs, manuals, etc.; ③ dissemination of the guideline in Chinese and English and the formation and dissemination of videos on websites; and ④ implementation of the guideline. The implementation of the guideline should be further promoted by publishing relevant interpretation articles of this guideline.

## 3 Guideline Recommendations

A total of seven clinical concerns, focusing mainly on the status, indications, contraindications, efficacy, combined application, use in special populations, adverse reaction response measures, and other clinical concerns of topical NSAIDs for the treatment of sports injuries, were selected in this guideline, and 22 recommendations were finally developed (Table 5).

**TABLE 5:** Guidelines for the use of topical NSAIDs in the treatment of sports injuries.

Clinical problem	Recommendations
1. What is the status of topical NSAIDs for sports injuries?	Recommendation 1: Topical NSAIDs are recommended as first‐line drugs for sports injuries given their high efficacy and safety (1B).
2. What are the indications and efficacy of topical NSAIDs for sports injuries?	Recommendation 2.1: Topical NSAIDs are recommended for OA pain management, especially in early and intermediate stage of knee and wrist OA (1A). Recommendation 2.2: Topical NSAIDs are recommended for pain relieve of the neck or back strain (1B). Recommendation 2.3: Topical NSAIDs are recommended for acute ankle sprain pain relive and reduce swelling (1B). Recommendation 2.4: Topical NSAIDs are recommended for the treatment of frozen shoulder and shoulder function improvement (1C). Recommendation 2.5: Topical NSAIDs are recommended for pain relive of lateral epicondylitis (1C). **Recommendation 2.6: Topical NSAIDs are recommended for tenosynovitis pain management (2C)**. **Recommendation 2.7: Topical NSAIDs are recommended for relieve tendinitis/tendinopathy pain (2C)**. Recommendation 2.8: Topical NSAIDs are recommended for synovitis treatment (2D).
3. What are the contraindications for topical NSAIDs in sports injuries?	**Recommendation 3.1: Topical NSAIDs are prohibited for skin wounds, rashes, and infected sites (1B)**. **Recommendation 3.2: Topical NSAIDs are prohibited for eyes or sensitive mucous membranes (1B)**. Recommendation 3.3: Before applying topical NSAIDs, details on the patient's allergy history should be obtained, and topical NSAIDs are prohibited for patients with allergies or asthma (1B).
4. What combination therapy options are available for topical NSAIDs in sports injuries?	Recommendation 4.1: Topical NSAID gels combined with therapeutic ultrasound are recommended to improve treatment efficacy (1B). **Recommendation 4.2: Topical NSAIDs combined with physical factors (e.g., ultrashort wave therapy, shock wave therapy, etc.) are recommended for sports injuries (2D)**. Recommendation 4.3: Topical NSAIDs are recommended for sports injuries combined with other drugs (2C)
5. Do topical NSAIDs require clinical pharmaceutical supervision for sports injuries?	Recommendation 5: Topical NSAIDs have high safety and effectiveness, meanwhile clinical pharmaceutical supervision and directed medication are recommended (1C).
6. Can topical NSAIDs be used in special populations with sports injuries?	Recommendation 6.1: The use of topical NSAIDs in children and elderly people is recommended to approach cautiously and monitor for adverse events (2C). Recommendation 6.2: Topical NSAIDs are not recommended during pregnancy and lactation (2D). Recommendation 6.3: The use of topical NSAIDs in patients with gastrointestinal disease is recommended to approach cautiously and monitor for adverse events (2D). Recommendation 6.4: The use of topical NSAIDs in patients with cardiovascular disease is recommended to approach cautiously and monitor for adverse events (2D). Recommendation 6.5: The use of topical NSAIDs in patients with liver and kidney dysfunction is recommended to approach cautiously and monitor for adverse events (2D).
7. What are the strategies for managing adverse events associated with topical NSAIDs for sports injuries?	Recommendation 7: When adverse reactions occur with topical NSAIDs in patients with sports injuries, the drug should be discontinued immediately. Drug washout should be performed, and adverse reactions should be treated (1B).

Clinical Question 1: What is the status of the use of topical NSAIDs for the treatment of sports injuries?

**Recommendation 1: Topical NSAIDs are recommended as first‐line drugs for the treatment of sports injuries given their high efficacy and safety (1B)**.

**Evaluation of evidence**: Topical NSAIDs are common drugs used for the treatment of sports injuries. Two systematic reviews (93 RCTs) were included to address this clinical issue. The GRADE evidence level is B. The questionnaire survey of clinically important questions revealed that patients and doctors have a high preference for use and that clinical application has more advantages than disadvantages, so the recommendation intensity is strong (1).

**Summary of evidence**: In 2015, Cochrane published a systematic review of topical NSAIDs for the treatment of acute musculoskeletal pain (61 RCTs in total), including topical NSAIDs such as diclofenac sodium, ibuprofen, ketoprofen, piroxicam, and indomethacin and formulations such as gel, emulsion, spray, and plaster. The results of the review revealed that topical NSAIDs provided more pain relief (pain reduction of 50 and above) for acute musculoskeletal pain than did the placebo [49]. In 2016, Cochrane published a systematic evaluation of topical NSAIDs for chronic musculoskeletal pain. Based on the systematic evaluation in 2012, 5 RCTs (32 RCTs in total) were included, and among them, 6 studies (2353 subjects) of topical diclofenac had a clinical success rate (NNT) of 9.8 [95% CI (7.1, 16.0)]. The NNT for topical ketoprofen gel was 6.9 [95% CI (5.4, 9.3)] in 4 studies (2573 subjects) [50].

Clinical Question 2: What are the indications and efficacy of the use of topical NSAIDs for the treatment of sports injuries?

**Recommendation 2.1: Topical NSAIDs are recommended for OA pain management, especially in the early and intermediate stages of knee and wrist OA (1A)**.

**Evaluation of evidence**: The use of topical NSAIDs is recommended for the treatment of OA pain on the basis of high‐quality systematic reviews and large‐sample RCTs. Three systematic reviews (91 RCTs) and two RCTs were included in this clinical question. The consistency of these studies in the literature is strong, the reliability of evidence integration of systematic evaluation is high, which is close to the clinical reality, and the evidence grade is A. The questionnaire survey of clinically important questions shows that patients and medical personnel have a high preference for use and that the advantages of clinical application obviously outweigh the disadvantages, so it is strongly recommended (1).

**Summary of evidence**: Deng et al. [51] published a meta‐analysis that included 9 RCTs (1333 in the treatment group and 1309 in the control group) on topical NSAIDs for OA treatment. Compared with those of the placebo, the safety, clinical pain relief rate, and potential functional improvement effect of topical diclofenac sodium were greater. Derry et al. [50] included 39 RCTs (10,857 patients with OA) in the Cochrane published systematic review. After 6–12 weeks of treatment, topical diclofenac and ketoprofen significantly relieved pain. In a systematic review published in 2018 that included 43 studies, namely, 36 RCTs (7900 OA patients) and 7 observational studies (218,074 OA patients), topical NSAIDs were found to reduce OA pain [95% CI (− 0.40, − 0.20)] and improve function [95% CI (−0.45, −0.24)] compared with the placebo. Among the topical NSAIDs included in the study, patients treated with the diclofenac patch had significantly less OA than did those treated with the placebo. Additionally, there was significant pain relief [SMD = −0.81, 95% CI (−1.12, −0.52)], and the piroxicam gel significantly improved function [SMD = −1.04, 95% CI (−1.60, −0.48)] [52]. In 2016, a multicenter RCT showed that the topical loxoprofen patch was comparable to oral loxoprofen in knee OA [53]. Additionally, in another RCT of elderly men and women with primary OA of the wrist, patients randomized to the topcoat 1% diclofenac sodium gel group (*n* = 198) or placebo group (*n* = 187), four times daily for 8 weeks, showed 42% to 45% reductions in their pain intensity scores and 36% to 40% reductions in their overall AUSCAN (Australian–Canadian osteoarthritis hand index) scores in the diclofenac sodium gel group [54]. Several domestic and international guidelines and consensuses also recommend the use of topical NSAIDs for the treatment of OA [19, 55–60].

**Recommendation 2.2: Topical NSAIDs are recommended for pain relief in the neck or for back strain (1B)**.

**Evidence evaluation**: For this clinical issue, 1 systematic review (of 23 RCTs) and 2 RCTs were included. There are many types of acute and chronic pain due to soft tissue injury to the neck and back. There are risk factors for research inaccuracy and publication bias in evidence quality ratings. The evidence rating is B. The results of a questionnaire survey of clinically important questions revealed that patients and medical staff have a high preference for use and that the advantages of clinical application are greater than the disadvantages, so it is strongly recommended (1).

**Summary of evidence**: Twenty‐three RCTs were included in the systematic review presented in the 2020 study on acute pain management (5170 participants). For acute pain, such as sprains, strains, and contusions, the NNTs for different topical NSAIDs compared with placebo were 4.7 [95% CI (3.7, 6.5)] for the diclofenac patch; 7.4 [95% CI (4.6, 19.0)] for the diclofenac patch plus heparin; and 1.8 [95% CI (1.5, 2.1)] for diclofenac gel [61]. In a study of 140 patients with acute lower back pain, all patients received intravenous dextromethorphan followed by 2 g of 2.5% ketoprofen gel or placebo at the sites of pain and tenderness. The pain relief at 30 min in the ketoprofen gel group was better than that in the placebo group. The rate of pain rescue drug use in the placebo group (10 patients, 14%) was significantly greater than that in the ketoprofen gel group (2 patients, 3%; *p* = 0.35) [62]. Predelet al. [63] included 72 patients with neck pain who were randomized 1:1 to diclofenac sodium cream (2 g, 1 day) for topical use. The visual analog scale (VAS; 100 mm) was used to evaluate motor pain and pain relief. The results showed that neck movement pain decreased by 10 mm after 1 h of treatment.

**Recommendation 2.3: Topical NSAIDs are recommended for acute ankle sprain pain relief and reduce swelling (1B)**.

**Evidence evaluation**: For this clinical issue, 1 systematic review (of 4 RCTs) and 2 RCTs were included. In the included studies, there were differences in acute ankle sprain severity, topical drug types, limited observation indicators, research limitations, and publication bias risk factors in terms of the grading of the quality of evidence. The evidence grade was B. The results of a questionnaire survey of clinically important questions revealed that patients and medical staff had a high preference for use and that the obvious advantages of clinical application were greater than the disadvantages, so it is strongly recommended (1).

**Summary of evidence**: A systematic review in 2014 of four RCTs of acute ankle sprain showed that topical NSAIDs (including ibuprofen, diclofenac, ketoprofen, and flurbiprofen formulations) were better for intravenous pain treatment at short‐term follow‐up [95% CI (−11.8, −1.2)] and for persistent pain treatment at mid‐term follow‐up [95% CI (−10.9, −3.0)] than the placebo. One of the RCTs evaluated weight pain, and the effect of NSAIDs on weight pain in the NSAID group was greater than that in the placebo group in the short‐ and medium‐term follow‐up [64]. In an RCT of acute ankle sprain in 2006, 2.5% ketoprofen gel or placebo was applied to a 5 cm area of the ankle sprain, and pain relief was measured via the VAS at 15 and 30 min. The study included 100 patients and revealed that ketoprofen gel provided more pain relief than did the placebo within 30 min and that the safety profile of ketoprofen gel was good [65]. In addition, studies have shown that oral or topical NSAIDs can effectively reduce short‐term pain and swelling in patients with ankle sprains and that topical NSAIDs do not increase the incidence of adverse events [66].

**Recommendation 2.4: Topical NSAIDs are recommended for the treatment of frozen shoulder and shoulder function improvement (1C)**.

**Evaluation of evidence**: One RCT was included for this clinical problem. This was a small‐sample study, different stages and layers of the frozen shoulder were not examined, and factors in this study are limited. The evidence grade is C. The questionnaire survey of clinically important questions shows that patients and medical personnel have a high preference for use and that the advantages of clinical application outweigh the disadvantages, so freezing treatment is strongly recommended (1).

**Summary of evidence**: This multicenter RCT included 158 patients who were randomized to receive DHEP lecithin gel (diclofenac gel component) or placebo (5 g each) for 10 days. The results showed that DHEP lecithin gel can effectively relieve pain and improve shoulder joint function in patients with frozen shoulders [67].

**Recommendation 2.5: Topical NSAIDs are recommended for pain related to lateral epicondylitis (1C)**.

**Evidence evaluation**: One systematic review (of 3 RCTs) was included to address this clinical issue. The existing studies did not stratify the severity of lateral epicondylitis, the types of topical drugs used were different, there were study limitations and publication bias risk factors, and the evidence grade was C. The results of the questionnaire survey of clinically important questions revealed that patients and medical personnel had a high preference for use and that the advantages of clinical application outweighed the disadvantages, so it is strongly recommended (1).

**Summary of evidence**: One systematic review including 3 RCTs with a total of 153 subjects revealed that topical NSAIDs were significantly more effective than placebo in the short term [95% CI (−2.42, −0.86)], with a clinical success rate of 7 [95% CI (3, 21)]. One of the studies (85 subjects) reported that after 14 days of treatment, more subjects reported good or excellent effects and fewer adverse reactions with topical NSAIDs than with placebo [RR = 1.49, 95% CI (1.04, 2.14)] [67, 68, 69].

**Recommendation 2.6: Topical NSAIDs are recommended for tenosynovitis pain management (2C)**.

**Evaluation of evidence**: One RCT was included to address this clinical question. There are many types of tenosynovitis, the existing research evidence is insufficient, there are research limitations and risks of bias, and the evidence grade is C. The results of a questionnaire survey of clinically important questions show that patients and medical staff have certain preferences for use and that clinical application may have more advantages than disadvantages, so topical NSAIDs for tenosynovitis are weakly recommended (2).

**Summary of evidence**: In this randomized controlled trial (RCT), the effects of tenosynovitis on the wrists of kayaking athletes were evaluated after randomization to the placebo or 1% diclofenac gel group. All participants received ice compression, stretching, and massage as standard treatments for tenosynovitis, were treated with diclofenac gel on day 1, and experienced pain relief on day 5 [70].

**Recommendation 2.7: Topical NSAIDs are recommended for relieving tendinitis/tendinopathy pain (2C)**.

**Evaluation of evidence**: Three RCTs were included to address this clinical question. There are many types of tendinitis/tendinopathy, the types of topical NSAIDs used vary, there are research limitations and risks of publication bias, and the evidence grade is C. The results of a questionnaire survey of clinically important questions revealed that patients and medical staff have certain preferences for use and that clinical application may have more advantages than disadvantages, so topical NSAIDs for tendinitis or tendinopathy pain are weakly recommended (2).

**Summary of evidence**: A multicenter RCT included 172 patients with tendinitis vulgaris treated with ketoprofen plaster once daily, and the pain relief rate was higher in the ketoprofen group (56%) than in the placebo group (37%) after 1 week of treatment (*p* = 0.001). The tolerability in both groups was satisfactory. The reported adverse events were mild localized allergic reactions on the skin, and these skin reactions gradually resolved spontaneously [71]. In an RCT of 30 patients with tendinitis published by Ginsberg et al. [72], each patient was treated with 4% indomethacin spray or a corresponding placebo 3–5 times daily for 14 days. After treatment with indomethacin spray, the treatment was effective in 80% of patients, and it was effective and well tolerated in 93% of patients. Bussin et al. [73] published an RCT that included 67 patients with persistent Achilles tendon disease who were randomly assigned to receive 10% topical diclofenac (*n* = 32) or placebo (*n* = 35) for 4 weeks. The mean Victorian Institute of Sports Assessment‐Achilles (VISA‐A) score improved significantly.

**Recommendation 2.8: Topical NSAIDs are recommended for synovitis treatment (2D)**.

**Evaluation of evidence**: One RCT and 2 observational studies were included to address this clinical question. Owing to study limitations and indirect evidence factors, the evidence grade is D. The results of the questionnaire survey of clinically important questions show that patients and medical personnel have certain preferences for use and that clinical application may have more advantages than disadvantages, so topical NSAIDs for synovitis are weakly recommended (2).

**Summary of evidence**: Jurca et al. [74] studied the rheumatoid synovium of 115 patients with rheumatoid arthritis treated with a combination of diclofenac sodium, methyl salicylate, and calendula extract gel formulation, a natural ingredient, and reported that the combination had good drug diffusion properties and provided increased tissue concentrations, resulting in improved and sustained pain relief and anti‐inflammatory effects. An animal study revealed high concentrations of topical NSAIDs in the synovium of knee joints in mice [75]. In a knee arthroscopic surgery study, peak plasma concentrations of topical NSAIDs were much lower than those of oral NSAIDs, and knee synovial drug concentrations were high, indicating high safety and efficacy [76].

Clinical Question 3: What are the contraindications for the use of topical NSAIDs in the treatment of sports injuries?

**Recommendation 3.1: Topical NSAIDs are prohibited for skin wounds, rashes, and infected sites (1B)**.

**Recommendation 3.2: Topical NSAIDs are prohibited for eyes or sensitive mucous membranes (1B)**.

**Recommendation 3.3: Before applying topical NSAIDs, details on the patient's allergy history should be obtained, and topical NSAIDs are prohibited for patients with allergies or asthma (1B)**.

**Evaluation and overview of evidence**: Regarding topical NSAIDs for the treatment of sports injuries, contraindications are the focus of attention in disease treatment, and the voter turnout rate in patient and medical staff surveys was 100%. There is no direct evidence of contraindications for the use of topical NSAIDs in the literature. The contraindications of topical NSAIDs are clearly indicated in the package inserts of chemical drugs (or corresponding topical NSAIDs) of the National Drug Administration (Table 1), so the evidence classification is B. Therefore, topical NSAIDS are strongly recommended for sports injuries (1).

Clinical Question 4: What combination therapy options are available for topical NSAIDs in sports injuries?

**Recommendation 4.1: Topical NSAID gels combined with therapeutic ultrasound are recommended to improve treatment efficacy (1B)**.

**Evidence evaluation**: Two controlled clinical studies were included to address this clinical issue. According to the 2016 edition, as a recommendation, the existing clinical effect is large, so the evidence grade has been revised to B. The investigation results show that medical staff prefer use and that the advantages of clinical intervention outweigh the disadvantages, so topical NSAIDs combined with therapeutic ultrasound are strongly recommended (1).

**Summary of evidence**: In 2022, in a clinical study published in 2000, the efficacy of ultrasound induction combined with NSAIDs in the treatment of sports injuries was evaluated but not confirmed, and the results revealed that pain relief among patients receiving treatment combined with ultrasound induction was more obvious [77]. In addition, in knee arthroscopic surgery patients, ketoprofen gel was applied externally before ultrasound treatment, and the results revealed that the drug concentration in knee synovial tissue was greater than that in adipose tissue after pulse and continuous ultrasound; moreover, the VAS score was significantly lower after treatment with diclofenac [78].

**Recommendation 4.2: Topical NSAIDs combined with physical factors (e.g., ultrashort wave therapy, shock wave therapy, etc.) are recommended for sports injuries (2D)**.

**Evaluation and summary of evidence**: A Chinese observational study of topical NSAIDs combined with physical factors in the treatment of sports injuries was included to address this topic. In this study, topical NSAIDs were combined with shockwave therapy for the treatment of chronic muscle pain, and the quality of the evidence was very low. After discussion with the expert panel, the evidence quality grade was classified as D. The results revealed that patients and medical personnel have a certain preference for use and that the clinical application may have more advantages than disadvantages; thus, this approach is weakly recommended. (2).

**Recommendation 4.3: Topical NSAIDs are recommended for sports injuries combined with other drugs (2C)**.

**Evaluation of evidence**: One systematic review (of 23 RCTs) and 1 RCT were included to address this clinical issue. In clinical practice, sports injury‐related conditions can be treated with other drugs according to the specific disease conditions. Topical NSAIDs are combined mainly with oral drugs (including oral NSAIDs, muscle relaxants, etc.), heparin, and sodium hyaluronate [79, 80, 81]. There are study inaccuracies and publication bias factors in the study of combination drugs, so the evidence grade is C. The investigation results show that patients and medical personnel have certain use preferences and that clinical application may have more advantages than disadvantages, so the combination of NSAIDs with other drugs is weakly recommended (2).

**Summary of evidence**: A systematic review assessed topical diclofenac. The therapeutic effect of acid plus heparin on various types of acute soft tissue pain was an NNT of 7.4 [95% CI (4.6, 19.0)] [61]. An RCT compared the efficacy and safety of oral and topical diclofenac combination therapy with those of topical or oral diclofenac monotherapy. There were no significant differences in pain relief, functional scores, or global health assessments between combination therapy and monotherapy, and the risk of adverse events was similar between the groups. Compared with oral NSAIDs alone, flurbiprofen patches combined with meloxicam tablets or etofenamate cream combined with sustained‐release ibuprofen capsules effectively reduce pain levels in OA patients without increasing the incidence of adverse events [80].

Clinical Question 5: Do topical NSAIDs require clinical pharmaceutical supervision for sports injuries?

**Recommendation 5: Topical NSAIDs are highly safe and effective in the treatment of sports injuries, but clinical pharmaceutical supervision and directed medication are recommended (1C)**.

Evidence evaluation and summary: There is no relevant evidence‐based research in the literature at present. Rational clinical drug use requires clinical pharmaceutical supervision and guidance, the clinical effect is large, and after discussion with the expert panel, the evidence quality is classified as C. Investigations of clinically important problems show that patients and medical personnel believe that clinical pharmaceutical supervision is needed. Clinical pharmaceutical supervision and guidance are necessary for older patients with multiple motor system injuries or other systemic diseases taking concomitant medications. Clinical pharmaceutical supervision and guidance include medication education, prescription reviews, medication consultation, etc. Clinical pharmaceutical supervision and guidance are helpful for optimizing drug regimens and reducing adverse reactions and medical costs. Patient medication awareness and drug compliance should be improved, and incorrect medication behavior should be avoided. In addition, clinical pharmaceutical supervision and guidance help identify at‐risk populations and early signs of serious adverse events to protect patients as much as possible. Clinical pharmaceutical supervision intervention measures have more advantages than disadvantages, so clinical pharmaceutical supervision is strongly recommended.

Clinical Question 6: Can topical NSAIDs be used in special populations with sports injuries?

**Recommendation 6.1: The use of topical NSAIDs in children and elderly people should be approached cautiously, and adverse events (2C) should be monitored**.

**Recommendation 6.2: Topical NSAIDs are not recommended during pregnancy or lactation (2D)**.

**Recommendation 6.3: The use of topical NSAIDs in patients with gastrointestinal disease should be approached cautiously, and adverse events (2D) should be monitored**.

**Recommendation 6.4: The use of topical NSAIDs in patients with cardiovascular disease should be approached cautiously, and adverse events (2D) should be monitored**.

**Recommendation 6.5: The use of topical NSAIDs in patients with liver and kidney dysfunction should be approached cautiously, and adverse events (2D) should be monitored**.

**Evaluation of evidence**: There is little direct research evidence on the use of topical NSAIDs in the treatment of sports injuries in special populations. There are research limitations and risks of occurrence bias, and the evidence rating is low. In the investigation of clinically important problems, the use of topical NSAIDs for sports injuries in special populations is of close concern. Topical NSAIDs have advantages over other routes of medication, so they are weakly recommended (2).

**Summary of evidence**: The use of topical NSAIDs for special populations with sports injuries is marked in the package inserts of chemical drugs (or corresponding topical NSAIDs) of the State Drug Administration (Table 1). There are clear age limits for the use of topical NSAIDs in pediatric patients, with one RCT of acute ankle sprain in children aged 7–18 years [82] and one Phase IV nonrandomized controlled clinical study of diclofenac in children aged 6–16 years [62]. The package inserts for topical NSAIDs clearly state that NSAID prescriptions for children should be given under the guidance of physicians and pharmacists. Elderly patients often have concomitant cardiovascular, liver and kidney, or gastrointestinal diseases, and NSAIDs are often used as first‐line treatments; therefore, these patients need to be closely monitored for adverse events [83, 84, 85, 86]. In pregnant and lactating patients, oral NSAIDs should be used only with a motor impairment assessment and systematic evaluation. There were 26 studies included in the review. A study of adverse reactions revealed that preterm birth during the second trimester of pregnancy was a major adverse event of indomethacin treatment [87, 88]. There are concerns about the use of NSAIDs while breastfeeding: some NSAIDs have antiplatelet effects and potential effects on neonatal platelet function. NSAIDs with COX‐1 and COX‐2 inhibitory effects have been reported to be contraindicated in breastfeeding and infants with thrombocytopenia or platelet dysfunction [88]. There is no direct evidence to evaluate the efficacy and safety of topical NSAIDs in patients with motor system injuries and gastrointestinal disorders. An analysis of data from 10 RCTs revealed that topical NSAIDs had a lower risk of gastrointestinal adverse events [RR = 0.66, 95% CI (0.56, 0.77), *p* < 0.001] and a lower risk of discontinuation [RR = 0.66, 95% CI (0.56, 0.77), *p* < 0.001] than did oral NSAIDs [0.84, 95% CI (0.68, 1.05), *p* = 0.13]. Topical NSAIDs are contraindicated in package inserts for patients with a history of active gastric ulcers/bleeding [89]. For cardiovascular events, oral NSAIDs are associated with increased cardiovascular risk but are still commonly used for pain management in patients with sports injuries [90]. In a cohort of clinical patients with rheumatoid arthritis, patients treated with topical NSAIDs had a lower risk of cardiovascular events than did patients treated with oral NSAIDs did [RR = 0.64, 95% CI (0.43, 0.95)], and patients treated with selective COX‐2 inhibitors had the highest risk of adverse cardiovascular reactions [91]. In patients with hepatic dysfunction, oral NSAIDs have been shown to have a low risk of liver injury (0.29/100,000), which may be associated with higher doses and long‐term treatment [92, 93]. High‐dose oral NSAID use has been associated with acute kidney injury, and chronic NSAID use has been associated with an increased risk of chronic kidney disease [94, 95] (Table 1).

Clinical Question 7: What are the strategies for managing adverse events associated with the use of topical NSAIDs for the treatment of sports injuries?

**Recommendation 7: When adverse reactions occur with the use of topical NSAIDs, the drug should be discontinued immediately. Drug washout should be performed, and adverse reactions should be treated (1B)**.

**Evaluation of evidence**: In the investigation of important clinical problems, the use of topical NSAIDs for the treatment of adverse events of sports injuries has always been a concern of patients and medical staff. The present study revealed that adverse reactions are comparable to those of the placebo, that adverse reactions are small, and that the drug should be discontinued immediately in the case of adverse reactions. The evidence grade is B. Researchers, patients and medical staff pay close attention to adverse reactions, and clinical intervention measures obviously have more advantages than disadvantages; therefore, these measures are strongly recommended. (1)

**Summary of evidence**: Few studies on adverse events following the use of topical NSAIDs for the treatment of sports injuries exist. In an RCT, the efficacy and tolerability of topical NSAIDs were evaluated in 30 patients with tendinitis and compared to those of a placebo. For each patient, indomethacin spray or placebo was applied 3 to 5 times daily for 14 days. The results revealed that only 2 patients experienced mild local skin irritation and did not need treatment interruption. Ninety‐three percent of patients tolerated it well [72]. Compared with those of placebo and oral NSAIDs, the risk of gastrointestinal adverse events, liver and kidney function damage, and cardiovascular events of topical NSAIDs was lower than that of oral NSAIDs and was equivalent to that of placebo in the study of topical NSAIDs for sports injuries. Topical NSAIDs have lower maximum plasma concentrations and longer maintenance times for local maximum drug concentrations [37]. A Cochrane systematic review of topical NSAIDs for acute pain published in 2015 included 5311 patients and reported mild localized adverse reactions in the skin, few systemic adverse events, or withdrawal from the trial due to adverse events [49]. According to the package insert of chemical drugs (or corresponding topical NSAIDs) of the State Drug Administration, especially for special groups, it is necessary to use drugs under the guidance of doctors or clinical pharmacists, avoid long‐term drug use, immediately stop the drug once adverse events occur, and actively address specific adverse reactions.

## 4 Discussion

This guideline provides evidence‐based recommendations for the treatment of sports injuries with topical NSAIDs to promote the rational use of NSAIDs. Topical NSAIDs are characterized by fast onset, high local concentrations, low systemic exposure, and few systemic adverse reactions. Compared with oral NSAIDs, topical NSAIDs are more suitable for the treatment of sports injuries, which is also one of the original goals of these guidelines. In the clinical practice process, we should fully consider the type of sports injury, the degree of injury, high‐risk groups (special groups and patients with comorbidities) and other factors and formulate a rational drug use plan. In this guideline, the recommendation is for NSAIDs to be used as prescribed under the guidance of clinicians and pharmacists, paying close attention to the selection of drug types and dosage forms, drug interactions, and the safety of topical NSAIDs and conducting health economic evaluations. This guideline is not intended to serve as a legal document. Clinical treatment options should vary from person to person under the constraints of individual patient conditions and clinical practice.

Moreover, this guide has certain limitations. The guidelines focus on topical NSAIDs, but for the treatment of sports injuries, clinically effective treatment methods also include rehabilitation physiotherapy, oral drug treatment, injection treatment, traditional Chinese medicine treatment, and surgical treatment, among others, and even a combination of multiple treatment methods. Owing to the limitations of the guidelines and the evidence presented in the literature, these guidelines do not provide additional recommendations. Guideline development is a systematic project, and guideline interpretation and suggestions are subsequently implemented. With developments in medicine and improvements in diagnostic and treatment processes, the contents of these guidelines will be further revised and improved.

## Conflicts of Interest

All the authors declare that they have no conflicts of interest to declare in the development of the guidelines. List of members of the Committee of Experts on the Development of Guidelines.

## Expert Groups for the Guideline

Guoping Li (Institute of Sports Medicine, General Administration of Sport of China), Shiyi Chen (Institute of Sports Medicine, Huashan Hospital Affiliated to Fudan University), Kunzheng Wang (Department of Orthopedic Surgery, Second Affiliated Hospital of Xi'an Jiaotong University), Jianquan Wang (Department of Sports Medicine, Third Hospital of Peking University), Chengqi He (Department of Rehabilitation Medicine, West China Hospital, Sichuan University), Jian Li (Sports Medicine Center, West China Hospital, Sichuan University).

## Expert Group on Guide Development

Yingfang Ao (Institute of Sports Medicine, Peking University), Gang Chen (Sports Medicine Center, West China Hospital, Sichuan University), Jian Chen (Department of Rehabilitation Medicine, Zhongshan Hospital, Xiamen University), Shiyi Chen (Institute of Sports Medicine, Huashan Hospital, Fudan University), Guoqing Cui (Department of Sports Medicine, Third Hospital, Peking University), Xiaofan Fei (Clinical Pharmacy Department, West China Hospital, Sichuan University), Chengqi He (Department of Rehabilitation Medicine, West China Hospital, Sichuan University), Hongchen He (Department of Rehabilitation Medicine, West China Hospital, Sichuan University), Chunyan Jiang (Department of Sports Medicine, Jishuitan Hospital, Beijing), Qing Jiang (Department of Sports Medicine and Adult Reconstructive Surgery, Drum Tower Hospital, Nanjing University Medical College), Guoping Li (Institute of Sports Medicine, General Administration of Sport), Jianjun Li (School of Rehabilitation Medicine, Capital Medical University), Jian Li (Sports Medicine Center, West China Hospital, Sichuan University), Pengcheng Li (Sports Medicine Center, West China Hospital, Sichuan University), Qi Li (Sports Medicine Center, West China Hospital, Sichuan University), Songcen Lv (Orthopaedics Department, Second Affiliated Hospital, Harbin Medical University), Yubin Luo (Rheumatology and Immunology Institute, West China Hospital, Sichuan University), Jinzhong Ma (Orthopaedics Department, First People's Hospital, Shanghai Jiaotong University), Xin Tang (Sports Medicine Center, West China Hospital, Sichuan University), Jianquan Wang (Sports Medicine Department, Peking University Third Hospital), Kunzheng Wang (Orthopedics Department, Second Affiliated Hospital, Xi'an Jiaotong University), You Wang (Orthopaedics Department, Ninth Affiliated Hospital, Shanghai Jiaotong University School of Medicine), Zhengzhen Wang (Sports Prescription Center, Beijing University of Sport), Xiaochun Wei (Orthopedics Department, Second Hospital, Shanxi Medical University), Ji Wu (Orthopedics Department, Air Force Medical Center), Gengyan Xing (Orthopedics Department, Third Medical Center, PLA General Hospital), Feng Ning (Department of Joint and Osteopathy, Dongfang Hospital Affiliated to Tongji University), Jiakuo Yu (Department of Orthopaedics and Sports Medicine, Beijing Tsinghua Changgung Hospital), Hui Zhan (Institute of Sports Medicine, General Administration of Sport), Weibin Zhang (Orthopedics Department, Ruijin Hospital Affiliated to Shanghai Jiaotong University School of Medicine), Mouwang Zhou (Department of Rehabilitation Medicine, Third Hospital, Peking University), Zongke Zhou (Orthopedics Department, West China Hospital, Sichuan University).

## Systematic Evaluation Group and Secretariat Working Group

Liang Du (Evidence‐Based Medicine Center, West China Hospital, Sichuan University), Yiquan Xiong (Evidence‐Based Medicine Center, West China Hospital, Sichuan University), Jiali Liu (Evidence‐Based Medicine Center, West China Hospital, Sichuan University), Weili Fu (Sports Medicine Center, West China Hospital, Sichuan University), Yujuan Li (Sports Medicine Center, West China Hospital, Sichuan University), Kexin Wang (Sports Medicine Center, West China Hospital, Sichuan University).

## External Review Expert Group

Wei Huang (Department of Orthopedics, First Affiliated Hospital of Chongqing Medical University), Jianhao Lin (Department of Orthopedics, People's Hospital of Beijing University), Tengbo Yu (Department of Orthopedics, Qingdao Municipal Hospital), Mingming Lei (Department of Sports Medicine, Affiliated Hospital of Chengdu University of Physical Education).

## Correspondence

Hongchen He (Department of Rehabilitation Medicine, Rehabilitation Key Laboratory of Sichuan Province, West China Hospital, Sichuan University), Guoping Li (Institute of Sports Medicine, General Administration of Sport of China), Shiyi Chen (Institute of Sports Medicine, Huashan Hospital Affiliated with Fudan University),Jian Li (Department of Orthorpedics and Sports Medicine,Orthopedic Research Institute, West China Hospital, Sichuan University).
